# Bayesian Hyper-LASSO Classification for Feature Selection with Application to Endometrial Cancer RNA-seq Data

**DOI:** 10.1038/s41598-020-66466-z

**Published:** 2020-06-16

**Authors:** Lai Jiang, Celia M. T. Greenwood, Weixin Yao, Longhai Li

**Affiliations:** 1 Lady Davis Institute for Medical Research, Jewish General Hospital, Montreal, Canada; 2 Department of Epidemiology, Biostatistics and Occupational Health, McGill University, Montreal, Canada; 3 Gerald Bronfman Department of Oncology, McGill University, Montreal, Canada; 4 Department of Statistics, University of California, Riverside, US; 5 Department of Mathematics and Statistics, University of Saskatchewan, Saskatoon, Canada

**Keywords:** Statistics, Cancer epigenetics, Cancer genomics, Gene expression

## Abstract

Feature selection is demanded in many modern scientific research problems that use high-dimensional data. A typical example is to identify gene signatures that are related to a certain disease from high-dimensional gene expression data. The expression of genes may have grouping structures, for example, a group of co-regulated genes that have similar biological functions tend to have similar expressions. Thus it is preferable to take the grouping structure into consideration to select features. In this paper, we propose a Bayesian Robit regression method with Hyper-LASSO priors (shortened by BayesHL) for feature selection in high dimensional genomic data with grouping structure. The main features of BayesHL include that it discards more aggressively unrelated features than LASSO, and it makes feature selection within groups automatically without a pre-specified grouping structure. We apply BayesHL in gene expression analysis to identify subsets of genes that contribute to the 5-year survival outcome of endometrial cancer (EC) patients. Results show that BayesHL outperforms alternative methods (including LASSO, group LASSO, supervised group LASSO, penalized logistic regression, random forest, neural network, XGBoost and knockoff) in terms of predictive power, sparsity and the ability to uncover grouping structure, and provides insight into the mechanisms of multiple genetic pathways leading to differentiated EC survival outcome.

## Introduction

The accelerated development of many high-throughput biotechnologies has made it affordable to collect complete sets of measurements of gene expressions. Scientists are often interested in selecting certain genes that are related to a categorical response variable, such as the onset or progression of cancer. These genes are known as *signatures* in the life sciences literature; for the purposes of our paper, we will call them *features*.

When finding features, we require an algorithm that can identify both sparse features and grouping structures. Sparsity is required because in the context of gene expression analysis, there are often only a few important features. Hence, the feature selection is expected to be sparse. Grouping structure is required because biological features often have an innate grouping structure. For example, there might be a high correlation between certain features; a group of genes might relate to the same molecular pathway, or be in close proximity in the genome sequence, or share a similar methylation profile^1,2^. To better understand disease etiology, therefore, one must understand the grouping structure of the genes associated with that disease.

At first, researchers concentrated on the sparsity problem in high dimensional feature spaces. They developed automatic sparse selection methods such as LASSO (Least Absolute Shrinkage and Selection Operator^3^) and knockoff variable selections^4,5^. In the Bayesian literature, LASSO is equivalent to a linear regression with (convex) Laplace penalty function on the coefficients. But for our purposes—namely, uncovering sparse features and grouping structure—the convex penalty functions have several problems. First, they are not sparse enough. Second, these functions are incapable of uncovering grouping structure. Indeed, the traditional convex sparse feature-selection algorithms are either unable to take grouping structure information into account, or else depend on prior knowledge of the specific grouping structure.

Again, some researchers focused on the first problem: the need for greater sparsity. One potential way to overcome this limitation is to amplify the signal. In recent years, researchers developed methods that are even more aggressive, and hence even more sparse, than LASSO. They proposed fitting classification or regression models with continuous non-convex penalty functions to discover features related to a response. Such non-convex penalty functions include, but are not limited to, hyper-LASSO^6^, global-local penalties^7^, *t* with small degrees of freedom^8,9^, SCAD^10^, horseshoe^11–15^, MCP^16^, NEG^17^, adaptive LASSO^18^, Dirichlet-Laplace and Dirichlet-Gaussian^19^, and generalized double-pareto^20^ functions. Reviews of non-convex penalty functions have been provided by^7,21,22^ and^23^. These non-convex penalties can shrink the coefficients of unrelated features (noise) to zero more aggressively than LASSO, while enlarging the coefficients of related features (signal).

These non-convex functions work well for sparsity; however, their effectiveness regarding grouping structure has not yet been explored. In fact, non-convex penalty will make selection within a group of highly correlated features: either splitting important features into different modes of penalized likelihood or suppressing less important features in favour of more important features. The within-group selection is indeed a desired property if our goal is selecting a sparse subset of features. Note that the within-group selection does not mean that we will lose other features within a group that are also related to the response because other features can still be identified from the group representatives using the correlation structure. On the other hand, the within-group selection results in a huge number of modes in the posterior (for example, two groups of 100 features can make 100^2^ subsets containing one from each group). Therefore, optimization algorithms encounter great difficulty in reaching a global or good mode because in non-convex regions, the solution paths are discontinuous and erratic.

The application of non-convex penalties in grouped feature selection remains limited by the computational difficulties, instead, researchers interested in grouping structure have tended to favor (convex) LASSO penalty. There have been three main approaches developed to consider directly the grouping structure in classification/regression models based on LASSO penalties:

1. The first approach is to develop methods that directly consider the grouping structure or the correlation among features in classification and regression models. This involves fitting classification models on the “new features” constructed from the feature groups (e.g., centroids or means) of features within groups; see^24–27^ and^28^.
2. The second approach is to fit classification models with penalties that enforce similarity on the coefficients of features within groups, such as group or fused LASSO^29,30^. Group and fused LASSO functions are more predictive than plain LASSO because they consolidate the predictive power of all features within groups.
3. The third approach is known as two-stage selection, and it includes supervised group LASSO (SGL)^31^ and ProtoLASSO^28^. SGL works in two stages: 1. apply LASSO to each group, separately, to determine the features of each group; and 2. apply LASSO to the selected features (from step one). ProtoLASSO, on the other hand, works as follows: 1. select prototype features within each group using marginal correlations; and 2. apply LASSO to the prototype features.

There are three more problems with all three of these approaches. First, the Lasso solution is subject to specific choices of regularization parameter *λ* and may not select the “correct” sparsity pattern in high dimensional data. For example, the solution may not be sparse enough when the signal to noise ratio is low. Second, these methods make selections separately in each group, so they cannot consider the joint effects of features from different groups. This is particularly limiting when analyzing biological interactions, because the individual associations of certain features (genes) with the outcome (disease) are low, but such features could still be useful when joined with other features (with a correlation structure). Third, in order to consider grouping structure, these methods require a pre-specified grouping index. This pre-specified grouping structure is often found via a clustering algorithm. However, the statistical algorithm’s results might not be a perfect match with meaningful feature groups (e.g., with biologically accurate clusters of genes). In addition, such grouping is probably too simple to explain complicated biological activities. For example, an outcome may be related to correlations among multiple groups of features—that is, interactions may occur both within and between groups.

Due to the numerous problems with convex functions, we hypothesize that non-convex functions would better suit our needs regarding sparsity and grouping structure. Unlike convex functions, non-convex functions have at least the potential to find grouping structures without prior knowledge. However, limited work has been done to take into account of grouping structure with non-convex penalties^32–34^. Moreover, non-convex methods are usually computationally expensive, which has limited their application to high-throughput biomedical data despite the potential usefulness.

Another problem is that the algorithms to solve these non-convex functions could be unstable^32^. When it comes to solving these non-convex penalty functions, the algorithms that provide solutions traditionally involve the process of *non-convex learning*. Specifically, non-convex learning processes are optimization algorithms for learning the classification and/or regression likelihood penalized by non-convex functions. But thus far, limited research has been done to develop stable optimization algorithm for non-convex function to uncover both sparse features and grouping structure. A review of such algorithms can be found in^32^ and^35^.

In this paper, we develop an approach to non-convex penalty functions, which we call BayesHL (for Bayesian Hyper-LASSO). BayesHL is a fully Bayesian approach that uses the Markov chain Monte Carlo (MCMC) method to explore the multi-modal posterior. This is a promising alternative to non-convex learning methods, because unlike many non-convex learning methods^32,36^, a well-designed MCMC algorithm can explore many modes to find multiple desirable feature subsets. The development of MCMC methods for exploring regression posteriors based on heavy-tailed priors has emerged only recently; the relevant articles include^9,37–41^, among others.

More specifically, we develop a sophisticated MCMC method to explore the posterior of a Robit model assigned with a class of heavy-tailed priors (i.e., Cauchy distribution with small scale). We employ the Hamiltonian Monte Carlo method^42^ to draw MCMC samples of the regression coefficients in a restricted Gibbs sampling framework. The framework’s computational complexity is more dependent on the number of signals than the number of features; this greatly accelerates the MCMC sampling efficiency for very high-dimensional problems. After MCMC sampling, we divide the samples into sub-pools according to the posterior modes to find a list of sparse feature subsets. This process is further aided by cross-validatory evaluation.

To the best of our knowledge, our work here is one of the few attempts to uncover grouping structure using MCMC in the context of a high-dimensional feature selection problem. The development of fully Bayesian (MCMC) methods for exploring regression posteriors based on heavy-tailed priors has emerged only recently; relevant articles include^6,9,37,43^.

Compared to other feature selection methods in the literature, BayesHL has two main benefits. First, BayesHL is more parsimonious than traditional convex (including LASSO) and non-convex learning methods. That is because BayesHL automatically makes selections within groups *without* a pre-specified grouping structure. Consequently, a single feature subset found by BayesHL is more parsimonious than the feature subsets selected by LASSO. It is also possible for BayesHL to consider the joint effects of features from different groups. Thus, the results always include representatives from different groups. Such succinct feature subsets are (compared to the results of traditional convex and non-convex methods) much easier to investigate and interpret according to existing biological knowledge. Second, BayesHL is immune to the problem of correlation bias. Other convex and non-convex methods may not be able to identify significant features when there is a high number of correlated features, due to the problem of correlation bias^2^. But BayesHL will always be able to identify the significant features because it enforces within-group selection, so even as the number of correlated features increases, the magnitudes of coefficients will not decrease.

Our MCMC-based non-convex learning method can effectively identify sparse feature subsets with superior out-of-sample predictive performance. This statement is based on the results of our experiment on a real high-throughput dataset of endometrial cancer. Results show that the BayesHL-selected feature subsets have better predictive power than those selected by competitors. Indeed, after further investigation we found that the BayesHL-selected gene subsets correspond to interactions between gene networks with meaningful biological interpretations.

In brief, in this paper we present a Bayesian feature subset selection method (BayesHL), test it, and use it to uncover an interesting result regarding endometrial cancer. We test our method on simulated datasets with independent or correlated groups of features, to demonstrate its feature subset selection and prediction performance with respect to grouping structures. We apply our method to a high-throughput dataset related to endometrial cancer, and present interesting findings of gene networks regarding the survival outcome of endometrial cancer.

## Methodology

### Model: heavy-tailed robit model

The following is an introduction to the notation we use throughout this paper. Suppose we have collected measurements of *p* features (such as genes) and a binary response (such as a disease indicator) on *n* training cases. For a case with index *i*, we use *y*_*i*_, taking integers 0 or 1, to denote the response value and use a row vector *x*_*i*_ to denote the *p* features, and the first element of *x*_*i*_ is set to 1 for including intercept term in linear model. Throughout this paper, we will use bold-faced letters to denote vectors or matrices. We will write collectively $y=({y}_{1},\ldots ,{y}_{n}){\prime}$, and $\,X=({x{\prime} }_{1},\,\ldots ,\,{x{\prime} }_{n}){\prime}$ in which rows stand for observations and columns stand for features. Note that, we use index 0 for the intercept term in this paper, ie., the values of the first column of *X* are all equal to 1, denoted by *x*_,0_. Using machine learning terminology, we call (*y*, *X*) training data, which are used to fit models; in contrast, the data used only in testing the predictive performance is called test data.

For the purposes of feature selection and binary classification, we are interested in modeling the conditional distribution of *y*_*i*_ given *x*_*i*_. The traditional **probit** models use a normally distributed auxiliary variable *z*_*i*_ to model *y*_*i*_ given *x*_*i*_ as follows:

1 $${y}_{i}=I({z}_{i} > 0),\,{z}_{i}={x}_{i}\,\beta +{\varepsilon }_{i},\,{\varepsilon }_{i} \sim N\mathrm{(0,}\,\mathrm{1)},$$

where *I*(·) is the indicator function, and *β* is a column vector of coefficients with the first element being intercept, denoted by *β*_0_. With *z*_*i*_ integrated out, the above model is equivalent to the following conditional distribution: $P({y}_{i}|{x}_{i},\,\beta )=\Phi {({x}_{i}\beta )}^{{y}_{i}}{(1-\Phi ({x}_{i}\beta ))}^{1-{y}_{i}},\,{\rm{for}}\,{y}_{i}=\mathrm{0,1,}$ where Φ is the cumulative distribution function (CDF) of the standard normal distribution.

In high-throughput data, there are typically a large number of extreme outliers. Since probit models cannot accommodate some extreme outliers (due to the light tails of normal distributions), we will use a more robust model: the Robit model^44^. Robit replaces the normal distribution for *ε*_*i*_ with a *t* distribution, and is thus more robust to outliers than probit and logistic regression (see^44,45^ and the references therein).

The Robit model is as follows:

2 $${y}_{i}=I({z}_{i}\, > \,\mathrm{0)}\,{z}_{i}={x}_{i}\,\beta +{\varepsilon }_{i},\,{\varepsilon }_{i} \sim T({\alpha }_{0},{\omega }_{0}),$$

where *T*(*α*, *ω*) stands for scaled student’s *t* distribution with degrees of freedom *α*, scale parameter $\sqrt{\omega }$, and mean parameter 0, with a probability density function (PDF) as ${t}_{\alpha ,\omega }(x)=\frac{\Gamma \left(\frac{\alpha +1}{2}\right)}{\sqrt{\alpha \pi }\,\Gamma \left(\frac{\alpha }{2}\right)}{\left(1+\frac{{x}^{2}}{\omega \alpha }\right)}^{-\frac{\alpha +1}{2}}\frac{1}{\sqrt{\omega }}$, where Γ(⋅) is the Gamma function. As in probit models, with *z*_*i*_ integrated out, the above model is equivalent to the following conditional distribution of *y*_*i*_ given *x*_*i*_:

3 $$P({y}_{i}|{x}_{i},\beta )={T}_{{\alpha }_{0},{\omega }_{0}}{({x}_{i}\beta )}^{{y}_{i}}{\mathrm{(1}-{T}_{{\alpha }_{0},{\omega }_{0}}({x}_{i}\beta ))}^{1-{y}_{i}},\,for\,{y}_{i}=\mathrm{0,1,}$$

where *T*_*α*,*ω*_(*x*) represents the CDF of *T*(*α*,*ω*).

*T*(*α*,*ω*) is given by ${T}_{\alpha ,\omega }(x)=\frac{1}{2}+\frac{\Gamma \left(\frac{\alpha +1}{2}\right)}{\sqrt{\alpha \pi }\Gamma \left(\frac{\alpha }{2}\right)}\times {}_{2}\,{F}_{1}\left(\frac{1}{2},\frac{\alpha +1}{2};\frac{3}{2};-\frac{{x}^{2}}{\alpha \omega }\right)\times \frac{x}{\sqrt{\omega }},$ where _2_*F*_1_ is the hypergeometric function, which is given as the sum of an infinite series^46^.

The *α*_0_ is fixed at *α*_0_ = 1, which is appropriate for modeling the possible range of outliers. In addition, from the CDF of the *t* distribution, we notice that only $\,\beta \,/\sqrt{{\omega }_{0}}$ is identifiable in the likelihood of (*ω*_0_, *β*) given observation *y*_1_, …, *y*_*n*_. Therefore, we fix *ω*_0_ at some reasonable value. We choose to use *ω*_0_ = 0.5 such that the ${T}_{{\alpha }_{0},{\omega }_{0}}$ is similar to the logistic distribution near origin zero, but has heavier tails (than the logistic distribution).

### Coefficient prior (*β*): heavy-tailed cauchy prior

Now that we have chosen a model for selecting features (Robit), the next step is to choose a framework for estimating *β*. The following subsections discuss why we chose to use a heavy-tailed (Cauchy) prior for estimating *β*, how we raised the sampling efficiency with restricted Gibbs sampling and Hamiltonian Monte Carlo, and why our MCMC algorithm is a good framework for obtaining an estimate of *β*.

In many problems of linking high-dimensional features to a response variable, it is believed that the non-zero regression coefficients are very sparse—that is, very few features are related to the response *y*. In the past decade, non-convex penalties have drawn the attention of many researchers because they can shrink the coefficients of unrelated features (noise) more aggressively to zero than the convex *L*_1_ penalty. In other words, non-convex penalties provide a *sharper* separation of signal and noise than *L*_1_.

In Bayesian methodologies, a non-convex penalty often corresponds to a prior distribution with a heavier tail than the Laplace distribution (which corresponds to *L*_1_). So in the Bayesian interpretation, a typical sample of *β* from a heavy-tailed prior has a few extraordinarily large values representing *related features*, and many small values representing *unrelated features*. Therefore, heavy-tailed priors are a better match for our expectations about *β* than the Laplace prior.

Of the suitable heavy-tailed priors, we have many choices, including Cauchy^8^, horseshoe^7,12–14,22,23^, and normal-exponential-gamma (NEG)^17^, all of which have been proven superior to *L*_1_ in detecting very sparse signals.

However, there are three reasons why we prefer Cauchy to horseshoe and NEG priors:

1. Although although the horseshoe and NEG priors have the same tail heaviness as Cauchy (converging to zero in the rate of 1/*β*^2^), they also have a non-differentiable log PDF at 0; therefore, if penalties are applied, small signals can be shrunken to exactly 0.
2. In our empirical comparison of the predictive performance of classification models using *t* priors with various tail heaviness (including NEG and horseshoe priors), we found that Cauchy had the optimal performance^6^.
3. Horseshoe and NEG priors demand additional computation in sampling the hyperparameters (the local variances for each *β*_*j*_, i.e., *λ*_*j*_ below). Indeed, in our aforementioned paper, the additional computation accounted for half of the whole sampling time, even after we used a restricted Gibbs sampling scheme to greatly shorten the sampling time for regression coefficients.

Therefore, we chose to use the plain Cauchy prior in this paper: *t* with degree of freedom *α*_1_ = 1, denoted by ${\beta }_{j} \sim T({\alpha }_{1},{\omega }_{1}),\,{\rm{for}}\,j=\mathrm{1,}\ldots ,p$.

For the purposes of MCMC sampling, we express the *t* prior for *β* as a scale-mixture normal by introducing a *latent* variance *λ*_*j*_ for each *β*_*j*_, as follows:

4 $${\beta }_{j}|{\lambda }_{j} \sim N\mathrm{(0,}{\lambda }_{j}),$$

5 $${\lambda }_{j} \sim {\rm{Inverse}}-{\rm{Gamma}}\left(\frac{{\alpha }_{1}}{2},\frac{{\alpha }_{1}{\omega }_{1}}{2}\right)\mathrm{}.$$

Hereafter, we will refer to this vector as *λ* = (*λ*_1_, …, *λ*_*p*_).

In order to shrink small coefficients toward zero, we must choose a very small-scale parameter $\sqrt{{\omega }_{1}}$ for Cauchy. In Bayesian methodologies, a typical way to avoid assigning a fixed value to a parameter is to treat it as a hyperparameter such that it will be chosen automatically during MCMC sampling according to marginalized likelihood. However, we have found that this approach does not choose at a sufficiently small scale to yield a very sparse *β*, because a classification model with *p* features can easily overfit a dataset with sample size $n\ll p$. In order to enforce sparsity in *β* and to improve the efficiency of MCMC sampling, we choose to fix $\sqrt{{\omega }_{1}}$ at a small value *e*^−5^ ≈ 0.01. Table 1 shows a number of upper-tailed quantiles of |*β*_*j*_| where ${\beta }_{j} \sim {\rm{Cauchy}}(0,{e}^{-5})$.

**Table 1:** Upper-tailed quantiles of absolute Cauchy with scale *e*^−5^.

Upper probability	0.200	0.100	0.020	0.010	0.002	0.001	0.0001
Quantile of |*β*_*j*_|	0.022	0.044	0.223	0.446	2.228	4.456	42.895

From Table 1, we see that this choice of value of *ω*_1_ postulates that 2 of the 1000 features have coefficients with magnitude ≥2.228. We believe that this is an appropriate level of sparsity for many high-dimensional feature-selection problems.

Another important reason to fix $\sqrt{{\omega }_{1}}$ is the “flatness” (heaviness) of a Cauchy tail. Due to this flatness, very small shrinkage is applied to large coefficients. Since the shrinkage is small, the estimates of large coefficients are robust to $\sqrt{{\omega }_{1}}$ ^6,13^. This is a distinctive property of priors with tails as heavy as Cauchy. (In other priors with similar tail heaviness, like Gaussian, Laplace priors, a careful choice of scale must be made because the shrinkage of large coefficients is large and sensitive to the scale.) Therefore, although $\sqrt{{\omega }_{1}}$ is fixed at a very small value around 0.01, the prior does not over-shrink large signals, and can accommodate a wide range of signals.

### Implementation: Estimating *β* using Restricted Gibbs Sampling with Hamiltonian Monte Carlo

There is great difficulty in maximizing the penalized likelihood function using heavy-tailed and small-scaled priors. For example, using a small scale for $\sqrt{{\omega }_{1}}$ such as *e*^−5^, the R function bayesglm in R package ARM (which implements penalized logistic regression with Cauchy priors) will converge to a mode where almost all coefficients are shrunken to very small values, even when the number of features (*p*) is small. On the other hand, using the default 2.5 value, bayesglm does not provide a sparse solution (to be presented in this article). The difficulty in optimization is further intensified by the severe multi-modality in the posterior because heavy-tailed and small-scaled priors can split coefficients of a group of correlated features into different modes rather than shrinking them simultaneously as Gaussian priors do. Therefore, although good theoretical properties of non-convex penalties have been proved in statistics literature (e.g.^16^), many researchers and practitioners have been reluctant to embrace these methods because optimization algorithms often produce unstable solutions^32^. This motivated us to develop MCMC algorithms for exploring the posterior with many modes due to the use of heavy-tailed priors.

Our MCMC algorithm will sample from the joint posterior, *f*(*β*, *λ*| *y*, *X*), which is based on the hierarchical models given by Eqs. (3–5) with *α*_0_, *ω*_0_, *α*_1_, *ω*_1_ fixed (so omitted in the following model descriptions). The log posterior can be written as follows:

6 $$\begin{array}{rcl}\log (f(\beta ,\lambda |y,X)) & = & \mathop{\sum }\limits_{i\mathrm{=1}}^{n}\,\log (P({y}_{i}|{x}_{i},\beta ))+\mathop{\sum }\limits_{j\mathrm{=0}}^{p}\,\log (f({\beta }_{j}|{\lambda }_{j}))\\  &  & +\mathop{\sum }\limits_{j\mathrm{=1}}^{p}\,\log (f({\lambda }_{j}))+C,\end{array}$$

where the first three terms come from the models defined by (3), (4), (5) respectively, and *C* is the log of the normalization constant unrelated to *β* and *λ*. The first three terms in (6) are given as follows:

7 $$\begin{array}{rcl}\log (P({y}_{i}|{x}_{i},\beta )) & = & {y}_{i}\,\log ({T}_{{\alpha }_{0},{\omega }_{0}}({x}_{i}\beta ))+\mathrm{(1}-{y}_{i})\log ({T}_{{\alpha }_{0},{\omega }_{0}}(-{x}_{i}\beta ))\\  &  & \equiv lp({y}_{i}|{x}_{i}\beta ),\end{array}$$

8 $$\log (f({\beta }_{j}|{\lambda }_{j}))=-\,\frac{1}{2}\,\log ({\lambda }_{j})-\frac{{\beta }_{j}^{2}}{2{\lambda }_{j}}+{C}_{1},\,{\rm{for}}\,j=\mathrm{0,}\ldots ,p$$

9 $$\log (f({\lambda }_{j}))=-\,\left(\frac{{\alpha }_{1}}{2}+1\right)\log ({\lambda }_{j})-\frac{{\alpha }_{1}{\omega }_{1}}{2{\lambda }_{j}}+{C}_{2},\,{\rm{for}}\,j=\mathrm{1,}\,\ldots ,\,p\mathrm{}.$$

where *C*_1_, *C*_2_ are two constants unrelated to (*β*, *λ*); the function *lp*(*y*_*i*_| *x*_*i*_*β*) is introduced to indicate that the probability of *y*_*i*_ given *x*_*i*_ is a function of *x*_*i*_*β*. An ordinary Gibbs sampling procedure to draw samples from (6) is to alternatively draw samples from the conditional posterior of *λ* given *β* with a log density equal to the sum of the last two terms of (6), and draw samples from the conditional posterior of *β* given *λ* with a log density equal to the sum of the first two terms of (6).

The challenge in sampling from the (6) comes from two aspects of high-dimensional features. One is the high dimension *p* of *β* (or *X*); the other is the high correlation among features *X*, which results in the high correlation in the conditional posterior of *β* given *λ*, and correspondingly the multi-modality in the marginal posterior of *β* (with *λ* integrated out). To combat these two difficulties, we propose an MCMC sampling algorithm that uses Gibbs sampling with Hamiltonian Monte Carlo (HMC) for sampling *β* in a restricted way. Our MCMC algorithm is sketched below and followed with explanations:

Starting from a previous state for (*β*, *λ*), a new state denoted by (*β*, *λ*) is obtained with these steps:

1. For each *j*, draw a new ${\hat{{\lambda }}}_{j}$ from the conditional distribution *f*(*λ*_*j*_|*β*_*j*_) with log PDF equal to the sum of (8) and (9). It is well-known that *λ*_*j*_ given *β*_*j*_ has an Inverse-Gamma distribution given as follows:
  10 $${\lambda }_{j}|{\beta }_{j} \sim {\rm{Inverse}}\,-\,Gamma\left(\frac{{\alpha }_{1}+1}{2},\frac{{\alpha }_{1}{\omega }_{1}+{\beta }_{j}^{2}}{2}\right)\mathrm{}.$$
2. With the new values of ${\hat{{\lambda }}}_{j}$ drawn in step 1, determine a subset, *β*_*U*_, of *β* to update in step 3 below. We update *β*_*j*_ if ${\hat{{\lambda }}}_{j}$ is large enough. That is, given a pre-scribed threshold value *η*, the subset is defined as $U=\{j|{\hat{{\lambda }}}_{j} > \eta \}$. The *β*_*U*_ is defined as {*β*_*j*_|*j* ∈ *U*}. The subset of *β*_*F*_ = {*β*_*j*_|*j* ∈ *F* = {0, …, *p*}\*U*} will be kept unchanged in step 3.
3. Update the set of *β*_*j*_ with *j* ∈ *U*, denoted by *β*_*U*_, by applying HMC to the conditional distribution of *β*_*U*_ given as follows:
  $$\log (f({\beta }_{U}|{\beta }_{F},\lambda ,X,y))$$
  11 $$=\mathop{\sum }\limits_{i\mathrm{=1}}^{n}\,lp({y}_{i}|{x}_{i,U}\,{\beta }_{U}+{x}_{i,F}{\beta }_{F})+\sum _{j\in U}\log (f({\beta }_{j}|{\hat{{\lambda }}}_{j}))+{C}_{3},$$
  where the function lp for computing log likelihood is defined in (7), and *x*_*i*,*U*_ is the subset of *x*_*i*_ with feature index in *U*. After updating *β*_*U*_, the new value of *β* is denoted by *β* in which *β*_*F*_ does not change. Note that, because HMC is a Metropolis algorithm, the new *β* may be equal to *β* if a rejection occurs.
4. Set (*β*, *λ*) = (*β*, *λ*), and go back to step 1 for the next iteration.

A typical sampling method for classification models is to augment a latent continuous value *z*_*i*_ for each categorical variable *y*_*i*_^47^, and sample from the joint distribution of *z*_1:*n*_ along with *β* and *λ* (e.g.^38^) with Gibbs sampling; we then can borrow algorithms developed for regression models with heavy-tailed priors^37,43^. Given *λ*_*j*_, the prior for *β*_*j*_ is a normal distribution. It is well-known that the posterior of *β* for normal regression given normal priors is a multivariate normal distribution with a covariance matrix involving *X*′ *X*. Note that this multivariate normal has a dimension *p*. When *p* is very large (e.g. thousands), drawing independent samples from a multivariate normal is extremely inefficient, because the required computation time for decomposing the covariance matrix will increase in the order of *p*^3^. Therefore, for drawing samples from *f*(*β*| *λ*, *X*, *y*), we choose to use Hamiltonian Monte Carlo (HMC), a special case of Metropolis-Hasting (M-H) algorithms, which explore the posterior in a local fashion without the need to decompose a high-dimensional matrix. HMC requires computing the log-posterior and its gradient. The gradient of log(*f*(*β*| *λ*, *X*, *y*)) given by the following expression:

12 $$\frac{\partial {\mathscr{U}}}{\partial {\beta }_{j}}=\mathop{\sum }\limits_{i\mathrm{=1}}^{n}\left[\frac{\varGamma \left(\frac{{\alpha }_{0}+1}{2}\right)}{\sqrt{{\alpha }_{0}\pi }\,\varGamma \left(\frac{{\alpha }_{0}}{2}\right)}\times \frac{{x}_{ij}}{\sqrt{{\omega }_{0}}}\times \frac{{\left(1+\frac{{({x}_{i,U}{\beta }_{U}+{x}_{i,F}{\beta }_{F})}^{2}}{{\alpha }_{0}{\omega }_{0}}\right)}^{-\frac{{\alpha }_{0}+1}{2}}}{1-{y}_{i}-{T}_{{\alpha }_{0},{\omega }_{0}}(x{}_{i,U}\,{\beta }_{U}+{x}_{i,F}\,{\beta }_{F})}\right]+\frac{{\beta }_{j}}{{\hat{{\lambda }}}_{j}},$$

where ${\mathscr{U}}$ is the function defined in (11). We can see that once the linear combination *Xβ* has been computed, the log posterior and its gradient can be obtained with very little computation. Computing *Xβ* is significantly cheaper than decomposing a matrix of dimension *p*. However, the random-walk behaviour of ordinary M-H algorithms limits the sampling efficiency of M-H algorithms. In HMC, the gradient of log posterior is used to construct a trajectory along the least constraint direction, therefore, the end point of the trajectory is distant from the starting point, but has high probability to be accepted; for more discussions of HMC, one is referred to a review paper by^42^.

From the above discussion, we see that obtaining the value of *Xβ* is the primary computation in implementing HMC. To further accelerate the computation for very large *p*, we introduce a trick called restricted Gibbs sampling; this is inspired by the fact that updating the coefficients with small *λ*_*j*_ (small prior variance in the conditional posterior of *β*_*j*_ given *λ*) in HMC does not change the likelihood as much as updating the coefficients with large *λ*_*j*_ but updating *β*_*j*_ with small or large *λ*_*j*_ consumes the same time. Therefore, we use *λ* in step 2 to select only a subset of *β*, denoted by *β*_*U*_, those have large prior variance *λ*_*j*_, to update in step 3 (HMC updating). We can save a great deal of time for computing *Xβ* in step 3 by caching values of *X*_*F*_*β*_*F*_ from the previous iteration because it does not change in the whole step 3; this greatly accelerates the construction of HMC trajectory. We typically choose *η* in step 2 so that only 10% of *β* are updated in step 3.

We clarify that although *β*_*F*_ (sometimes the whole *β*) are kept the same in an iteration, the choice of *U* in step 2 for the next iteration will be updated because *λ* will be updated in step 1. Thus, *β*_*j*_ will not get stuck to a very small absolute value, unlike that in optimization algorithms this typically occurs.

The above restricted Gibbs sampling is a valid Markov chain transition for the joint posterior (6). To understand this, let us recall that, in Gibbs sampling we can arbitrarily choose any variables to update with a Markov chain transition that leaves the conditional distribution of chosen variables invariant, provided that the choice of variables to be updated does not depend on the *values* of the chosen variables in the previous iteration. For example, it is not a valid Markov chain transition if we choose *β*_*j*_ with large |*β*_*j*_| in the previous iterations; by contrast, it is a valid Markov chain transition if we choose *β*_*j*_ to update by referring to variances of *β*. In step 3, the choice of *β*_*U*_ does not depend on the values of *β* in the previous step. Instead, the choice only depends on the value of *λ*_*j*_ in the previous step, which partially determines the variances of *β* in $f(\,\beta \,|\hat{{\lambda }},\,X\,,\,y\,)$. Therefore, the updates of *β*_*U*_ in step 3 is reversible with respect to $f(\,\beta \,|\hat{{\lambda }},\,X\,,\,y\,)$.

The advantage of HMC is that it can explore highly correlated posterior quickly with a long leapfrog trajectory without suffering from the random-walk problem. This ability of HMC also plays an important role in travelling quickly between multiple modes of the posterior. This is explained as follows. When ${\hat{{\lambda }}}_{j}$ and ${\hat{{\lambda }}}_{k}$ for two correlated features *j* and *k* are large after a draw in step 1, the joint conditional posterior of (*β*_*j*_, *β*_*k*_) given $({\hat{{\lambda }}}_{j},{\hat{{\lambda }}}_{k})$ are highly negatively-correlated. For such distributions, HMC can move more quickly than random-walk algorithms along the least constrained direction, and this move will lead to the change of modes in joint distribution of (*β*_*j*_, *β*_*k*_) with *λ* integrated out.

There are a huge number of modes in the posterior even when *p* is moderate when there are a large number of correlated features. In the empirical studies reported in this paper, we use a two-stage procedure. In **Stage 1**, we run the restricted Gibbs sampling with HMC using the dataset containing all *p* features. Then we calculate MCMC means of all coefficients *β* and choose only the top p* = 100 features with largest absolute values of MCMC means. The stage 1 is very time consuming. In **Stage 2** we re-run the MCMC sampling with only the selected features once again. Our feature selection will be based on the MCMC samples obtained from Stage 2. A list of setting parameters with recommended values for ease in reference are given in the Supplementary Information.

### Feature subset selection: MCMC sample subdivision

**Figure 1:**
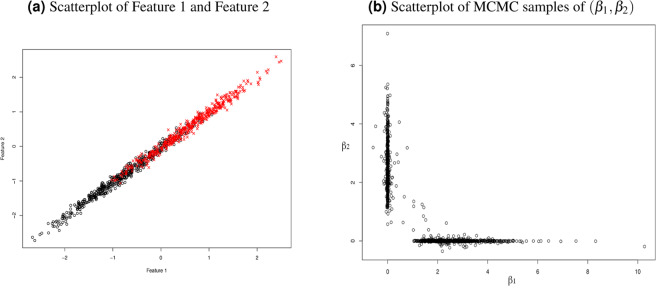
Demonstration of within-group selection with two correlated features for binary response. Color denotes the response value for each case. Note that the two features together do not provide significantly more information than only one for classifying the response.

We run the MCMC sampling to obtain samples from the posterior of *β*. With the intercept *β*_0_ removed, this sample is denoted by a matrix *B* = (*β*_*j*,*i*_)_*p*×*R*_, in which *β*_*j*,*i*_ represents the value of *β*_*j*_ in the *i* th sample and *R* is the number of MCMC samples. The posteriors of *β* for Robit models with heavy-tailed priors are severely multi-modal. For a demonstration, one can look at Fig. 1, which shows a scatterplot of MCMC samples of two *β*_*j*_’s for two correlated features. Therefore, we should divide the whole Markov Chain samples *B* into sub-pools according to the mode that each sample represent. However, the number of such feature subsets may be huge even the number of features *p* is small. Therefore, we only consider dividing Markov Chain samples according to the multiple modes for the Markov Chain samples obtained in **Stage 2** in which a large number of weakly related features have been omitted. In this article, we use a scheme that looks at the relative magnitude of *β*_*j*_ to the largest value in all features. The scheme is rather ad-hoc. However, it is very fast and works well in problems of moderate dimension, such as *p* = 100. More advanced methods for collecting feature subsets from MCMC is our priority for future research. The scheme used in this article is described as follows:

1. We set *I*_*j*,*i*_ = 1 if |*β*_*j*,*i*_| > 0.1 × max{|*β*_1,*i*_|, …, |*β*_*p*,*i*_|}, and *I*_*j*,*i*_ = 0 otherwise. By this way, we obtain a boolean matrix (*I*_*j*,*i*_)_*p*×*R*_ with its entry *I*_*j*,*i*_ denotes whether the *j* th feature is selected or not in *i* th sample.
2. Discard the features with overall low frequency in step 1. We calculate ${f}_{j}=\frac{1}{R}\mathop{\sum }\limits_{i\mathrm{=1}}^{R}{I}_{j,i}$. We will discard a feature *j* if *f*_*j*_ is smaller than a pre-defined threshold, which is set to be 5% as an ad-hoc choice in this article. Let *D* = {*j*|*f*_*j*_ < 5%}. For each *j* ∈ *D*, we set *I*_*j*,*i*_ = 0 for all *i* = 1, ..., *R*. This step is to remove the features that come into selection in step 1 due to MCMC randomness.
3. Find a list of feature subset by looking at the column vectors of *I*. Each unique column in *I* represents a different feature subset.

The above algorithm is not the best for dividing the MCMC samples according to the posterior modes of *β*. The reason is that the MCMC simulation introduces small jitters into the *β*_*j*_’s of the features not selected in the mode. The above algorithm aims to get rid of the jitters by using thresholding in step 1 and step 2. However, they may not eliminate some jitters. This will result in some feature subsets with very small frequency. The optimal algorithm may be to find the posterior modes starting from each MCMC sample using a certain optimization algorithm. However, finding the posterior modes for a large number of MCMC samples is time-consuming. In this paper, we present the results based on the above fast algorithm for simplicity. From our informal comparisons, the results are fairly close to the feature subsets found by hunting the mode from each MCMC sample.

### Predictive performance metrics

The frequencies of feature subsets in MCMC samples may not exactly reflect their predictive power. In this paper, we evaluate the predictive power of each feature subset using leave-one-out cross-validation (LOOCV) using the same training cases for simulating MCMC. Specifically, for each collected feature subset, we apply logistic regression model with *t* penalty^8^ and evaluate its predictive performance using 4 criteria: **error rate**, average of minus log predictive probabilities (**AMLP**), the area under ROC (receiver operating characteristic) curve (**AUROC**) and the area under precision-Recall curve (**AUPRC**). AMLP is calculated at each actually observed *y*_*i*_: $\frac{1}{n}\,{\sum }_{i\mathrm{=1}}^{n}\,-\,\log ({\hat{P}}_{i}({y}_{i}))$, and it punishes heavily the small predictive probabilities at the true class labels. We use an R-package pROC^48^ to compute AUROC and AUPRC. We propose three prediction methods based on BayesHL MCMC sampling results. The prediction methods based on the *top* feature subset with the highest posterior frequency, and the *optimal* feature subset (with the smallest cross-validated AMLP) are referred, respectively, as **BayesHLtop** and **BayesHLopt**. We will refer to the result by averaging the predictive likelihood over MCMC samples as the default **BayesHL** method.

We prefer AMLP and AUPRC as better metrics (than AUROC and error rate) for evaluating model performance in our study. In fact, AUROC values are not informative when (i) data is imbalanced with few cases of the minority class^49^ (e.g. high risk patients) or (ii) the cost of misclassifying the minor class (high risk patients) is more of concern^50^. Under scenario (i), AUPRC is usually preferred over AUROC because the latter can be misleading in such cases and provide deceptively optimistic results^51^. AMLP (i.e. estimate of entropy^52,53^) is a better choice than AUROC under both scenarios (i) and (ii), since the former incorporates “certainty” of classification directly in the calculation. In general, AMLP is a more scalable metric than AUROC and AUPRC. It also penalizes severely misclassifications and thus favors more robust methods. In contrast, these misclassifications have less influence on AUROC and AUPRC, but may carry significant costs in applications.

### Overview: BayesHL

In summary, we proposed a heavy-tailed Robit model with heavy-tailed Cauchy priors for coefficients *β* to explore the potential useful posterior modes. The model (*β*) is then estimated using our adapted restricted Gibbs sampling method with Hamiltonian Monte Carlo technique. Finally, multiple feature subsets, corresponding to multiple posterior modes, were collected from the MCMC samples and used to perform classification problems. This method is referred to as the Bayes Hyper Lasso method **BayesHL**. The prediction performance of **BayesHL** will be tested and compared to other methods in simulation analysis and a gene expression data analysis.

## Simulation Studies

### An example with independent groups of features

In this section, we compare BayesHL with other existing feature selection methods on simulated datasets with independent groups of features. Each dataset has *p* = 2000 features and *n* = 1200 cases, 200 of which are used as training cases and the other 1000 cases are used as test cases. With *z*_*ij*_, *ε*_*ij*_, *e*_*i*_ generated from *N*(0, 1), we generate the feature values *x*_*ij*_ for *i* = 1, ..., *n*, *j* = 1, ..., *p* in four groups and the class label *y*_*i*_ as follows:

13 $${x}_{il}={z}_{i1}+0.5{\varepsilon }_{il},i=\mathrm{1,}\,\mathrm{...,}\,n,l=\mathrm{1,}\,\mathrm{...,}\,\mathrm{50,}\,({\bf{Group}}\,{\bf{1}})$$

14 $${x}_{im}={z}_{i2}+0.5{\varepsilon }_{im},i=\mathrm{1,}\,\mathrm{...,}\,n,m=\mathrm{51,}\,\mathrm{...,}\,\mathrm{100,}\,({\bf{Group}}\,{\bf{2}})$$

15 $${x}_{ik}={z}_{i3}+0.5{\varepsilon }_{ik},i=\mathrm{1,}\,\mathrm{...,}\,n,k=\mathrm{101,}\,\mathrm{...,}\,\mathrm{150,}\,({\bf{Group}}\,{\bf{3}})$$

16 $${x}_{ij} \sim N\mathrm{(0,1),}i=\mathrm{1,}\,\mathrm{...,}\,n,j=\mathrm{151,}\,\mathrm{...,}\,\mathrm{2000,}\,({\bf{Group}}\,{\bf{4}})$$

17 $${y}_{i}=1\,{\rm{if}}\,({z}_{i1}+{z}_{i2}+{z}_{i3})/\sqrt{3}+0.1{e}_{i} > \mathrm{0;}\,=0\,{\rm{otherwise}}.$$

The *z*_*i*1_, *z*_*i*2_ and *z*_*i*3_ are common factors for features in respective group. Since the features within each of Group 1–3 are related to a common factor, they are highly correlated. However, the features across groups are independent. The response *y*_*i*_ is generated with the values of the common factors *z*_*i*1_, *z*_*i*2_, *z*_*i*3_. Therefore, *y*_*i*_ is related to all the features in Group 1–3. The *y*_*i*_ is unrelated to all the features in Group 4. The true model of *y*_*i*_ given *x*_*ij*_ has non-zero coefficients for all features in Group 1–3.

We apply BayesHL and other methods including LASSO, Group LASSO (GL), supervised Group LASSO (SGL), Random Forest (RF), Penalized Logistic Regression (PLR) with hyper-LASSO penalty, neural network (NN^54^), eXtreme Gradient Boosting (XGBoost^55^), and knockoff variable selection method (Knockoff^4^) to fit the training cases and then test their performance with the 1000 test cases. The implementation details of all the competitors can be found in the supplement. BayesHL is conducted with the default parameter settings as listed in supplement. We run BayesHL first with all 2000 features in **stage 1**, and then rerun with p* = 100 top features selected with posterior means, both with the aforementioned settings. The feature selection and prediction use the MCMC samples in the **stage 2** with the top 100 features. Because of the large *p* in stage 1, we ran BayesHL hours to ensure convergence. We allowed BayesHL to run about 30 minutes to obtain the results reported throughout this article.

Table 2 shows the top (by frequency) five feature subsets selected by BayesHL. According to the AMLP, the top feature subset (1,57,140) is identified as the optimal feature subset too. We see that the top 4 feature subsets selected by BayesHL contain exactly one feature from each of Group 1–3 (each with 50 features) and none from Group 4 (noise).

**Table 2:** Top 5 feature subsets selected by BayesHL, and their within-sample leave-one-out cross-validatory predictive power. “fsubsets” gives I.D. of features in each subset, “coefs” is the vector of regression coefficients found with the posterior means, “AMLP” - “AUPRC” are cross-validatory predictive power measures of each feature subset.

	fsubsets	freqs	AMLP	ER	AUROC	AUPRC
1	1,57,140	0.22	0.13	0.09	0.99	0.99
2	1,51,140	0.11	0.13	0.08	0.99	0.99
3	16,57,140	0.10	0.14	0.08	0.99	0.99
4	1,51,101	0.09	0.14	0.08	0.99	0.99
5	12,57	0.04	0.41	0.39	0.89	0.90

We also compare the out-of-sample predictive power of the top and optimal feature subset found by BayesHL with the “complete” feature subsets selected by other methods. We compare 4 predictive measures (ER, AMLP, AUROC, AUPRC) against the number of features used in making predictions, as shown in Table 3. The numbers of features used in BayesHLtop and BayesHLopt are the number of features in the top and optimal subsets. To count the number of features selected by the methods other than BayesHL, if automatic sparse selection is not available, we threshold their absolute coefficients by 0.1 of the maximum to decide whether or not they are used in predictions. We choose 0.1 as a threshold because we use the same thresholding to obtain the top and and optimal feature subsets of BayesHL.

**Table 3:** Comparison of feature selection and out-of-sample prediction performance of different methods on a dataset with independent group of features. The number of features used by the others other than BayesHL are counted after thresholding the absolute coefficients by 0.1 times the maximum. BayesHLopt: optimal feature subset from BayesHL. BayesHLtop: top feature subset from BayesHL. BayesHL: average prediction probability across feature subsets identified by BayesHL. RF: random forest. NN: neural network. XGboost: eXtreme Gradient Boosting. Knockoff: knock off variable selection followed with logistic regression. AMLP: average minus log-probabilities. AUROC: area under ROC. AUPRC: area under precision-recall curve.

**(a) Numbers of selected features in respective group**											
	BayesHLtop	BayesHLopt	BayesHL	LASSO	GL	SGL	RF	PLR	NN	XGBoost	Knockoff
Group 1	1	1	4	6	49	7	49	50	50	32	49
Group 2	1	1	4	5	50	10	49	50	50	0	50
Group 3	1	1	3	6	50	6	48	50	50	0	50
Group 4	0	0	0	13	341	12	14	1305	1252	0	16
Total	3	3	11	30	490	35	160	1455	1402	32	165
**(b) Out-of-sample predictive performance**											
ER	0.10	0.10	0.06	0.09	0.07	0.10	0.08	0.08	0.10	0.34	0.10
AMLP	0.22	0.22	0.15	0.21	0.22	0.24	0.38	0.18	0.20	0.63	0.31
AUROC	0.97	0.97	0.99	0.97	0.99	0.97	0.98	0.98	0.98	0.75	0.97
AUPRC	0.97	0.98	0.99	0.96	0.98	0.98	0.98	0.97	0.98	0.75	0.97

Table 3, demonstrates that the BayesHL has the best predictive performance, which is better than the best performer in non-BayesHL methods—Group LASSO with more than 490 features. Specifically, BayesHL achieved the lowest ER (0.06), lowest AMLP (0.15), highest AUROC (0.99) and AUPRC (0.99) among all methods. The values of ER, AUPRC and AUROC stay at fairly similar levels for all methods except XGBoost. Only AMLP values (i.e. cross-entropy estimates^52,53^) are substantially different across methods and BayesHL outperforms others on this metric. BayesHLtop and BayesHLopt have slightly worse predictive performance than non-BayesHL methods, however, they use only 3 features, one from each signal group. In terms of efficiency in selecting useful features, BayesHLtop and BayesHLopt do the best jobs if we look at the ratio of predictive measure to number of used features. In comparison, other methods are all less sparse and select much larger subsets from noise group (Group 4). Particularly, Group LASSO enforces the similarity of coefficients in each group, therefore, all the features in signal groups along with a large number (341) of noise features are selected.

### An example with correlated weakly differentiated features

In this section we will compare the performance of BayesHL in a simulated scenario such that two groups of features are weakly differentiated but have a strong joint effect on the response. Specifically, a dataset with *n* = 1200 cases and *p* = 2000 features is generated as follows:

18 $$P({y}_{i}=c)=\frac{1}{2},\,{\rm{for}}\,c=\mathrm{1,2,}$$

19 $${x}_{ij}={\mu }_{{y}_{i}\mathrm{,1}}+{z}_{i1}+0.5{\varepsilon }_{ij},\,{\rm{for}}\,j=\mathrm{1,}\,\mathrm{...,}\,\mathrm{200,}\,({\bf{Group}}\,{\bf{1}})$$

20 $${x}_{ij}={\mu }_{{y}_{i}\mathrm{,2}}+0.8{z}_{i1}+0.6{z}_{i2}+0.5{\varepsilon }_{ij},\,{\rm{for}}\,j=\mathrm{201,}\,\mathrm{...,}\,\mathrm{400,}\,({\bf{Group}}\,{\bf{2}})$$

21 $${x}_{ij}={\mu }_{{y}_{i}\mathrm{,3}}+{z}_{i3}+0.5{\varepsilon }_{ij},\,{\rm{for}}\,j=\mathrm{401,}\,\mathrm{...,}\,\mathrm{600,}\,({\bf{Group}}\,{\bf{3}})$$

22 $${x}_{ij} \sim N\mathrm{(0,1),}\,{\rm{for}}\,j=\mathrm{601,}\,\mathrm{...,}\,\mathrm{2000,}\,({\bf{Group}}\,{\bf{4}})$$

where *z*_*ij*_ and *ε*_*ij*_ are from *N*(0, 1), and the means of features in Group 1–3 in two classes are given by the following matrix *μ*_*c*,1:3_, where *μ*_1,1:3_ = (−0.3,0.3,1) and *μ*_2,1:3_ = (0.3, −0.3, −1).

A dataset generated as above has 200 features in each of Group 1–3 related to the response and the remaining 1400 are completely noisy. Each feature in Group 1 and Group 2 is weakly differentiated due to the small difference in class means (0.3 vs −0.3). The features within each group are positively correlated with correlation 0.8. Additionally, a feature from Group 1 and a feature from Group 2 has a correlation coefficient 0.64 because they share a common factor *z*_*i*1_. Therefore, a combination of two features from Group 1 and 2 respectively has clear joint effect for *y*_*i*_.

We run BayesHL and other methods to a dataset generated as above and use 1000 test cases to compare the out-of-sample predictive power of the top and also optimal feature subset with the “complete” feature subsets found by other methods using the same procedure for obtaining Table 4. Table 4 shows BayesHL methods have slightly worse predictive performance than Group LASSO and PLR, which successfully combine the power of all signal features from Group 1–3 to make better predictions. However, the ROC curves of Group LASSO (AUROC = 0.97) and BayesHL (AUROC = 0.95) are not significantly different under Delong’s two-sided test^56^ (*p*-value = 0.47). Moreover, the feature subsets selected by Group LASSO and PLR are significantly less sparse and include many noise features in Group 4, while BayesHL, LASSO, SGL and RF have more sparse results. Particularly, BayesHL selected 6, 8, and 6 features respectively from each of the three signal groups, which demonstrated the clear strength of our method in sparsity and interpretability. In conclusion, BayesHL delivered similarly good prediction performance as its competitors, while using much more sparse feature subsets.

**Table 4:** Comparison of feature selection and out-of-sample prediction performance of different methods on a simulated dataset with correlated weakly differentiated features. BayesHLopt: optimal feature subset from BayesHL. BayesHLtop: top feature subset from BayesHL. BayesHL: average prediction probability across feature subsets identified by BayesHL. RF: random forest. NN: neural network. XGboost: eXtreme Gradient Boosting. Knockoff: knock off variable selection followed with logistic regression. AMLP: average minus log-probabilities. AUROC: area under ROC. AUPRC: area under precision-recall curve.

**(a) Numbers of selected features in respective group**											
	BayesHLtop	BayesHLopt	BayesHL	LASSO	GL	SGL	RF	PLR	NN	XGboost	Knockoff
Group 1	1	1	6	3	155	4	3	172	200	136	36
Group 2	1	1	8	3	123	5	5	177	200	0	9
Group 3	1	1	6	7	176	12	102	192	200	0	170
Group 4	0	0	1	10	215	22	3	1020	1259	0	17
Total	4	3	21	23	669	43	113	1561	1859	136	232
**(b) Out-of-sample predictive performance**											
ER	0.18	0.17	0.12	0.14	0.10	0.16	0.15	0.10	0.10	0.14	0.43
AMLP	0.47	0.48	0.33	0.34	0.25	0.46	0.37	0.26	0.31	0.35	16.88
AUROC	0.90	0.91	0.95	0.93	0.97	0.92	0.93	0.95	0.92	0.92	0.55
AUPRC	0.91	0.92	0.94	0.93	0.96	0.95	0.93	0.96	0.92	0.92	0.55

## Endometrial Cancer RNA-Seq Data Analysis

Endometrial cancer (EC) starts in the cells of the inner lining (endometrium) of the uterus. It is one of the most common cancers of the female reproductive system and is particularly common in women over age 60.

We chose to analyze the endometrial cancers from The Cancer Genome Atlas (TCGA) Research Network (http://cancergenome.nih.gov/) since there is a large number of tumour samples with matched gene expression profiles and clinical information. The original TCGA-EC dataset contains around 500 samples of EC with matched gene expression profiles and clinical information. This is one of the largest samples in TCGA’s database.

We obtained TCGA data from the Broad GDAC Firehose (using bioconductor Rpackages TCGAbiolinks and TCGA2STAT), which includes *N* = 269 samples with matched RNASeq profile and clinical information, after filtering steps such as restricting to primary solid tumor as sample type and endometrioid endometrial adenocarcinoma as histological type. We further filtered 3 patients with missing radiation information. The *N* = 266 matched RNASeq profiles were downloaded in RPKM (Reads Per Kilobase Million)-normalized format and log2-transformed. We then performed univariate feature selection and retained *P* = 7298 (out of 20502) genes with high values of coefficient of variation (≥5), or high values of mean expression of log2 RPKM (≥3).

The rest of this section is organized as follows: we first compare the predictive performance of our algorithm vs. the competitors with a classification analysis on 5-year EC survival outcome, then introduce and discuss the results of survival analysis and pathway analysis. Finally, we discuss possible biological explanations for the results of these analyses.

### Comparison of the algorithms’ results via classification analysis

We applied all methods to the dataset (*n* = 266 samples, *p* = 7298 features), with leave-one-out cross-validation, to perform binary classification for 5-year overall survival (OS) outcome of patients (**Y**). Specifically, classification methods were trained on each leave-one-out training set (265 samples) and then used to predict on the leave-out test case. Covariates such as age, diagnosis year and radiation therapy are also included in the model. The cross-validated prediction probabilities across folds were then collected to evaluate the overall performance for all methods. For the purpose of comparison, we performed the same steps detailed above with all algorithms: LASSO, Group LASSO (GL), supervised Group LASSO (SGL), Random Forest (RF), Penalized Logistic Regression (PLR) with a hyper-LASSO penalty, neural network (NN), eXtreme Gradient Boosting (XGBoost), knockoff (knockoff) variable selection, and BayesHL. Note that we report the prediction probabilities from BayesHL as explained in the method section. The implementation details of other methods can be found in the supplement.

Table 5 presents the results of our analysis. As can be seen, BayesHL had the best performance out of all the classifiers with respect to three of the four measures (false predictions, AMLP, and area under precision-recall curve (AUPRC)). GL demonstrated slightly better predictive performance than BayesHL with respect to area under ROC (AUROC). However, the differences of ROC curves from BayesHL and Group LASSO are not statistically significant under Delong’s two-sided test^56^ (*p*-value = 0.79). The AUPRC values are substantially different across methods and BayesHL provide the optimal result. BayesHL also have the optimal AMLP values. Note that the infinite AMLP values from the Random Forest and Neural Network methods imply that there exist extreme misclassifications. Finally, both Group LASSO and SGL performed relatively better than the rest of methods (except BayesHL), suggesting potential existence of grouping structures among genes. In conclusion, we prefer AMLP and AUPRC (over AUROC and ER) to evaluate the performance of all methods for this imbalanced classification problem (12.8% high risk patients), and under both metrics our BayesHL method clearly demonstrated better prediction power by using more succinct feature subset selections.

**Table 5:** Comparison of cross-validated predictive performance on endometrial cancer data (N = 266) using all methods. AMLP: average minus log probabilities. AUROC: area under ROC, AUPRC: area under precision-recall curve. GL: Group LASSO, SGL: supervised Group LASSO, RF: Random Forest, PLR: Penalized Logistic Regression with a hyper-LASSO penalty, NN: neural network, XGBoost: eXtreme Gradient Boosting, knockoff: knockoff variable selection followed with logistic regression.

	BayesHL	LASSO	GL	SGL	RF	PLR	NN	XGBoost	knockoff
False Predictions	29	35	33	32	34	49	55	37	39
AMLP	0.30	0.34	0.31	0.32	Inf	0.98	Inf	0.34	0.60
AUROC	0.78	0.72	0.80	0.79	0.73	0.65	0.70	0.77	0.70
AUPRC	0.54	0.31	0.41	0.42	0.36	0.25	0.29	0.35	0.32

### Comparison via survival analysis

**Figure 2:**
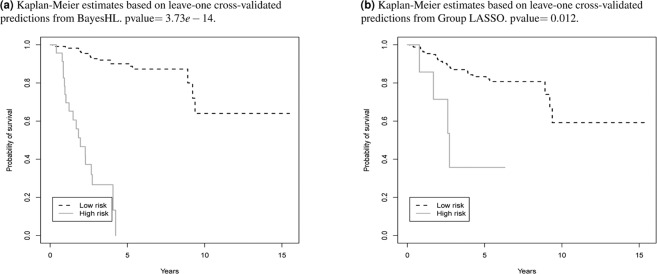
Kaplan-Meier estimates of the 5-year OS for all (266) EC patients, according to the cross-validated prediction probabilities from BayesHL and Group LASSO.

Next, we compared the algorithms’ results by performing a Kaplan-Meier correlation. The 5-year OS for each patient was calculated using leave-one-out cross-validation. Patients were classified as either high-risk or low-risk according to the minimum survival probability, with 0.5 as the cutoff threshold. The Kaplan-Meier curve in Fig. 2 shows that patients in the high-risk group had significantly lower 5-year OS than those in the low-risk group (log-rank test *p* = 3.73*e*−14) based on the BayesHL predictions. By comparison, Group LASSO did not achieve statistical significance *p* = 0.012 in the same Kaplan-Meier test. Note that we only compare the results of BayesHL with Group LASSO here because of their superior classification performance in Table 5.

### Comparison via IPA pathway analysis

**Figure 3:**
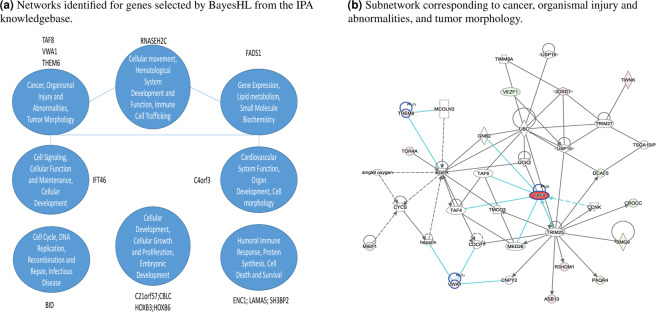
Molecular network information of the genes selected by BayesHL from the IPA knowledgebase. (**a**) All networks identified for genes selected by BayesHL. (**b**) Subnetwork corresponding to cancer, organismal injury and abnormalities, and tumor morphology. Arrows with solid lines represent direct interactions and arrows with broken lines represent indirect interactions. Direction of the arrows represents causal effects from upstream to downstream or protein self-bindings. The shapes of blocks correspond to different classes of general molecular functions in IPA knowledgebase. The color inside each block reflects the (averaged) gene coefficients from BayesHL model. Red indicates that the expression of the gene has negative impact on survival outcome and cyan indicates positive impact. White denotes no impact. The blocks with blue circle and green edges denote genes that occur in the selected 19 feature subsets from BayesHL. Both figures were generated through the use of IPA^57^ software (QIAGEN Inc., https://www.qiagenbioinformatics.com/products/ingenuity-pathway-analysis).

Finally, we conducted a pathway analysis of the identified gene signatures using the Ingenuity Pathways Analysis (IPA) system^57^. The resulting network is derived from the Ingenuity Knowledge Base and indicates which roles the input genes play in a global molecular network. The purpose of the IPA analysis is to see whether the genes output by our method and the competitors are related to potentially cancer-linked subnetworks. Figure 3a shows the results of our IPA analysis for the BayesHL-selected features on the whole dataset. As can be seen, the BayesHL-selected features are related to eight subnetworks, one of which is clearly linked to a cancer outcome (“Cancer, organismal injury and abnormalities, and tumor morphology”).

### Explanation of the results

In this section, we examine the genes selected by BayesHL and explore why some of these genes might be linked to EC survival outcomes. Table 6 provides a list of the BayesHL-selected feature subsets, along with measures of their leave-one-out cross-validated predictive performance on the whole dataset.

**Table 6:** BayesHL-selected feature subsets and the corresponding cross-validated prediction performance on the endometrial cancer dataset (N = 266). AMLP: average minus log probabilities. AUROC: area under ROC, AUPRC: area under precision-recall curve.

	frequency	Genes	False Prediction	AMLP	AUROC	AUPRC
1	0.1	FADS1, TAF8	28	0.33	0.73	0.45
2	0.08	HOXB6, TAF8	29	0.32	0.75	0.44
3	0.07	CBLC, TAF8	31	0.34	0.72	0.42
4	0.07	SH3BP2, TAF8	32	0.33	0.75	0.45
5	0.07	C4orf3, TAF8	28	0.30	0.78	0.51
6	0.06	BID, TAF8	31	0.33	0.76	0.42
7	0.06	C21orf57, TAF8	30	0.32	0.76	0.43
8	0.06	FADS1	34	0.39	1.00	0.07
9	0.05	SH3BP2	35	0.37	0.66	0.28
10	0.05	C8orf55, ENC1	34	0.37	0.63	0.24
11	0.05	IFT46, TAF8	33	0.31	0.81	0.45
12	0.04	HOXB3, TAF8	28	0.32	0.75	0.48
13	0.04	C8orf55	34	0.37	0.64	0.17
14	0.04	RNASEH2C, TAF8	30	0.34	0.72	0.43
15	0.04	LAMA5	33	0.36	0.65	0.25
16	0.04	C4orf3, HOXB3, TAF8	25	0.29	0.81	0.56
17	0.03	TAF8, VWA1	32	0.32	0.75	0.45
18	0.03	C21orf57	33	0.36	0.67	0.29
19	0.03	C4orf3, FADS1, TAF8	27	0.31	0.77	0.51

We observe that genes HOXB3 and SH3BP2 both appear twice in Table 6. This is supporting evidence for our claim of BayesHL’s effectiveness, because these genes are known to be prognostic markers for endometrial cancer. HOXB3 can induce the transformation and proliferation of tumor cells in breast cancer^58^ and ovarian cancer^59^. It has recently been tested as a regulation target to control endometrial cancer^60^. The protein encoded by SH3BP2 functions in the cell that signals various immune response pathways^61^. This information, combined with our finding (that SH3BP2 might be a survival-related gene for EC patients) suggest that EC cells may induce an abnormal immune system response that leads to a worse survival outcome. For example, we know that SH3BP2 protein helps to regulate signaling pathways that activate B cells and macrophages, whose infiltration in necrosis in the tumor center (hot-spot tumor associated macrophages) is a hazard factor to relapse-free survival of endometrial cancer patients^62^.

From Table 6, we also observe that the gene TAF8 appears numerous times. Moreover, TAF8 plays a central role in the cancer-related subnetwork we found during our IPA analysis (see Fig. 3b below for a detailed view of how BHTF-selected genes fit into the cancer and morphology subnetwork). Specifically, BayesHL assigns gene TAF8 the highest coefficient value, and the IPA analysis located TAF8 in one of the central positions in the cancer and morphology subnetwork (highlighted in red in Fig. 3b). It is known that TAFs contribute to the differentiation and proliferation of cells, and several TAFs (including TAF2, TAF4B, TAF9) have been identified as tumor promoters or suppressors in ovarian cancer^63,64^. However, more research must be done before we can establish a link between TAF8 and EC; and as of yet, no study exists on that topic.

In conclusion, these results suggest a potential central role of TAF8, VWA1 and THEM6 in the endometrial cancer development and survival outcome, by their repeated occurrence in most of feature subsets identified by FBRHT. Another interesting network identified by IPA is the one associated with cellular development and growth, proliferation and embryonic development, which includes C21orf57, CBLC, HOXB3, and HOXB6. Our finding that the representatives from these two sub-networks are repeatedly selected by FBRHT may suggest that the development of endometrial cancer, as well as the corresponding survival outcome, could be influenced by the regulation factors of cell proliferation and pathways of protein binding process. In conclusion, this study identified several candidate genes and sub-networks that may play an important role in key aspects of endometrial cancer development, and eventually lead to different survival outcome.

## Discussion

In this paper, we proposed a feature selection method, Bayesian Robit regression with Hyper-LASSO priors (BayesHL), that employs MCMC to explore the posteriors of Robit classification models with heavy-tailed priors. We conducted experiments— with real data— that demonstrate BayesHL’s ability to find sparse feature subsets with good predictive power, and to automatically make selections within groups of correlated features (without a pre-specified grouping structure). In future work, we would like to improve the accuracy of our feature subset selection method, and apply our Bayesian Inference framework to other models and non-convex penalties.

Regarding the first goal, we hope to optimize the selection of feature subsets. Currently, MCMC introduces small random jitters into the sample values of a *β*_*j*_, which inadvertently lead to the selection of certain undesirable features. To address this problem, we use a large arbitrary threshold of 0.1 (on the relative magnitudes of coefficients) to eliminate such undesirable features. But this results in overly sparse feature subsets and risks omitting features with small coefficients. Future work should aim to resolve this optimization problem without introducing over-sparsity. There are three general approaches we could take. First, we could consider a fast optimization algorithm, which can take the sparsity in coefficients into consideration, to find the exact modes from the MCMC samples. Second, we could use mixture modeling or clustering methods to divide MCMC samples according to their modes, and third, we could use a “reference approach” to find the feature subset (from among the MCMC samples) that gives similar predictions as the global mode of all the MCMC samples (not the best within-sample predictive power)^65^.

Finally, another possible direction for future work is to apply the Bayesian inference we developed in this paper to many other models (e.g., linear, graphical) and non-convex penalties to address feature selection problems in different application domains.

In conclusion, we would like to highlight two interesting findings from this study. First, our experiments with high-dimensional data— show that BayesHL results are comparable, in terms of their predictive power, to those of competitors (including LASSO, group LASSO, supervised group LASSO, random forest, penalized logistic regression, neural network, XGBoost and Knockoff) using far more sparse feature subsets. Secondly, in verifying the efficacy of BayesHL, we not only uncovered sparse feature subsets; we also identified genes that may be biologically meaningful in determining the survival outcome of endometrial cancer patients. Although we know that much work remains to be done, our results demonstrate that BayesHL has enormous potential for use in gene expression analysis.

## Supplementary information

Supplementary Information.
