# Supplementary material for: Bayesian Hyper-LASSO Classification for Feature Selection with Application to Endometrial Cancer RNA-seq Data
Source: Sci Rep. 2020 Jun 16;10:9747. doi: 10.1038/s41598-020-66466-z (PMC7297975; doi:10.1038/s41598-020-66466-z)
Supplement: Supplementary file 1 — Supplementary Information. [file 41598_2020_66466_MOESM1_ESM.pdf]

A Details of Computational Methods

A.1 Setting Parameters of BayesHL

We run MCMC sampling in two stages. In stage 1, we run MCMC sampling with all p features. Then we use MCMC means of p coefficients to choose the top p^* features. In stage 2, we run MCMC sampling with a reduced dataset with only the p^* selected features from stage 1. Typically, we use the same MCMC sampling settings in two stages as listed below.

- Model specification parameters: $\alpha_0, \omega_0, \alpha_1, \omega_1$

  The α_0 and $\sqrt{\omega_0}$ are the shape and scale parameter of t distribution for modelling $y_i|\boldsymbol{x}_i$ in (3equation.0.3). The α_1 and $\sqrt{\omega_1}$ are the shape and scale parameter of t distribution as the prior for β_j. They are all fixed at $\alpha_0 = 1, \omega_0 = 0.5, \alpha_1 = 1, \omega_1 = \exp(-10)$ in most experiments if there is not specific mentioning.

- Restricted Gibbs sampling thresholding η

  In step 3 of "Restricted Gibbs sampling with HMC" presented in Sec. equation.0.4, we only choose β_j with $j \in U = \{j|\hat{\lambda}_j > \eta\}$ to update with HMC. We typically choose η so that 10% of $\boldsymbol{\beta}$ are updated.

- HMC step size adjustment factor ϵ and lengths of trajectory l_1 and l_2

  There are two critical tuning parameters for HMC: the step size of each leapfrog step and the length of leapfrog trajectory. Fortunately they can be tuned independently [4]. Following [4], we set leapfrog step size ϵ_j for β_j with the second order derivative multiplied by a common adjustment factor ϵ: $\epsilon_j = \epsilon \left(\frac{\partial^2 \mathcal{U}}{\partial \beta_j^2}\right)^{-1/2}$. The ϵ is an adjustment factor usually chosen from 0.1 to 1 such that we obtain the optimal rejection rate 30% for HMC [4]. The required second-order derivative of $\mathcal{U}$ with respect to β_j is approximated by: $\frac{\partial^2 \mathcal{U}}{\partial \beta_j^2} \approx \sum_{i=1}^{n} \frac{x_{ij}^2}{\hat{\lambda}_j} + \frac{1}{\hat{\lambda}_j}$, where x_{ij} are the value of the jth feature in the ith case.

  The choice of length of trajectory is a little complicated. [3] recommended to run HMC in two phases: initial (burn-in) phase and sampling phase. In initial phase, one uses a leapfrog trajectory of short length l_1 so that the log likelihood can be changed more quickly and the Markov chain can more quickly reach equilibrium or a local mode for our problems. In sampling phase, one should use a leapfrog trajectory of longer length l_2 to make full use of the ability of HMC to reach a distant point from the starting. l_2 is usually chosen after some pre-run experiments. Users may want to pre-run a Markov chain with a relatively large value of l_2 (e.g. 500,1000) and look at the trajectory of the magnitude of $\boldsymbol{\beta}$. Because the leapfrog trajectory may go backwards to the starting point, l_2 should be chosen such that the magnitude of $\boldsymbol{\beta}$ is explored in only one direction to the furthest extent without backtracking. However, the optimal choice of l_2 is hard. It depends on specific problems. In addition, for our problems, the posterior are highly multi-modal, therefore, the optimal choice of l_2 may vary for

different modes. An automatic scheme for choosing l_2, called NUTS, is proposed by [2].

In our empirical studies, for the simplicity, we use $l_2 = 50$ which appears sufficiently long for our problems. We set a shorter $l_1 = 10$ in burn-in phase for faster convergence. We note further that it is possible to avoid this ad-hoc selection of leap-frog steps by adopting NUTS algorithm instead of plain HMC. This points to some avenues for future research through investigations of computational efficiency to improve the performance of our method.

A.2 Implementation of Existing Feature Selection Methods

- Penalized Logistic Regression using Hyper-LASSO penalty (PLR)

  We use the function `bayesglm` in the R package `arm` to fit Penalized Logistic Regression using Hyper-LASSO penalty. The function `bayesglm` uses the penalty based on $T(\alpha, \omega)$ prior, the scaled t-distribution with shape parameter α and scale parameter $\sqrt{\omega}$. By default, `bayesglm` sets $\alpha_1 = 1$ and scale parameter $\sqrt{\omega_1} = 2.5$ after the feature values are standardized in the suggested way [1].

- LASSO

  LASSO is implemented using the R package `glmnet`. The choice of regularization parameter λ is critical for the performance of LASSO. We feed the R function `glmnet` with a set of regularization parameters $\lambda = \{\lambda_m, m = 1, 2, ..., M\}$. By default, we start with minimum λ_1 value $\lambda_1 = 0.01$ and choose $M = 100$ candidate values with $\lambda_m = 0.01m, m = 1, 2, ..., M$. To find an optimal LASSO solution, we conduct cross-validation with respect to average minus log-probability over all candidate λ_m values. At last, we rerun `glmnet` on the whole dataset again with the optimal λ.

- Group LASSO (GL)

  We implement Group LASSO with prior group structure determined by hierarchical clustering (HC). We first conduct hierarchical clustering with the `hclust` function in the R package `clust` on the feature matrix X. For a given number of groups C, the R function `hclust` can construct a tree with UPGMA (Unweighted Pair Group Method with Arithmetic Mean), and then the tree is cut into several groups by specifying the desired number of groups C. The optimal value of C is chosen using the maximum silhouette value from the set of $\{2, \ldots, 50\}$. With a chosen group structure (index), we can run Group LASSO (using the R function `gglasso`) on different values of the regularization parameter λ. An optimal λ is chosen to minimize the cross-validated AMLP (average minus log-probability). At last we fit Group LASSO again with this optimal λ and the given group structure.

- Supervised Group LASSO (SGL)

  We use the same group structure as used for Group LASSO. Given this group structure, SGL is implemented with a two-stage strategy. In stage 1, for each feature group we then implement the LASSO algorithm with a reduced dataset and use the LASSO solution to extract significant features. More specifically, we fit LASSO (as we introduced

before) with all the features in the kth group. The features with nonzero coefficients in the resulting LASSO solution will be retained and used as representatives of group k. In stage 2, all group representative features are then combined into a consolidated training dataset, with their group indices being retained. We then fit Group LASSO as described above on this consolidated dataset with the retained group indices.

- Random Forest (RF)

  We implement Random Forest algorithm with the R package `RandomForest` (based on Breiman and Cutlers original Fortran code). Two important parameters in Random Forest are the number of trees (`ntree`) to grow and the number of variables randomly sampled as candidates at each split in the forest (`mtry`). With two arbitrary sets of candidate values for them, we fit randomForest with cross-validation. By default we use the candidate values of `mtry` ranging from $\sqrt{p}$ to n if $\sqrt{p} < n$, or n to $\sqrt{p}$ if $\sqrt{p} > n$. The candidate values of `ntree` are chosen from 250 to 500. For each pair value of `mtry` and `ntree` we run the Random Forest algorithm with the R function `randomForest` with cross-validation. The optimal pair values of `mtry` and `ntree` are then selected with respect to minimum AMLP. We then fit the whole dataset again with the optimal value of `mtry` and `ntree`.

A.3 An Investigation of Computational Efficiency of BayesHL

In this section, we use a simulation experiment to briefly demonstrate the high efficiency of the sampler used in BayesHL. We will focus on the efficiency of BayesHL sampler in exploring multiple modes of Robit posterior distributions, by comparing to the JAGS [5], a black-box MCMC sampler. JAGS cannot scale well for very high-dimensional problems. Therefore, we simulate a dataset with only $p = 100$ features for this comparison. Such examples also represent the stage-2 of BayesHL in which only a pre-selected small feature subset is used. However, we want to point it out that BayesHL works well in very high-dimensional problems such as with $p = 5000$ in our real data analysis, except that the results are harder to interpret due to the large number of feature subsets. We generate a dataset consisting of 4 groups of features. 10 features are in each of Group 1-3, and 70 features in Group 4. We run BayesHL and JAGS with Robit model for a long time (16835 seconds). For BayesHL, we use the same settings as given in the appended Section A.1. We thin the original MCMC iterations into 1000 super-transitions in a way such that each super-transition (a transition consists of multiple original iterations) in BayesHL and JAGS costs the same time. We divide the 1000 MCMC samples produced by both BayesHL and JAGS to find feature subsets using the same way as described in Section equation.0.12. We then monitor whether a mode switching occur from two consecutive super-transitions. From our experience BayesHL sampler is much more efficient than JAGS in exploring a large number of modes of Robit posterior distributions.

B Gene Networks Identified by LASSO and RandomForest for Endometrial Cancer

Figure S1: Networks identified for endometrial cancer from LASSO selection by Ingenuity Pathways Analysis. (a). Networks identified for genes selected by LASSO from the IPA knowledgebase. (b) Subnetwork corresponding to Cell Cycle, DNA Replication, Recombination, and Repair, Cell-To-Cell Signaling and Interaction. Red indicates that the expression of the gene has negative impact on survival outcome and cyan indicates positive impact. White denotes no impact.

(a) (b)

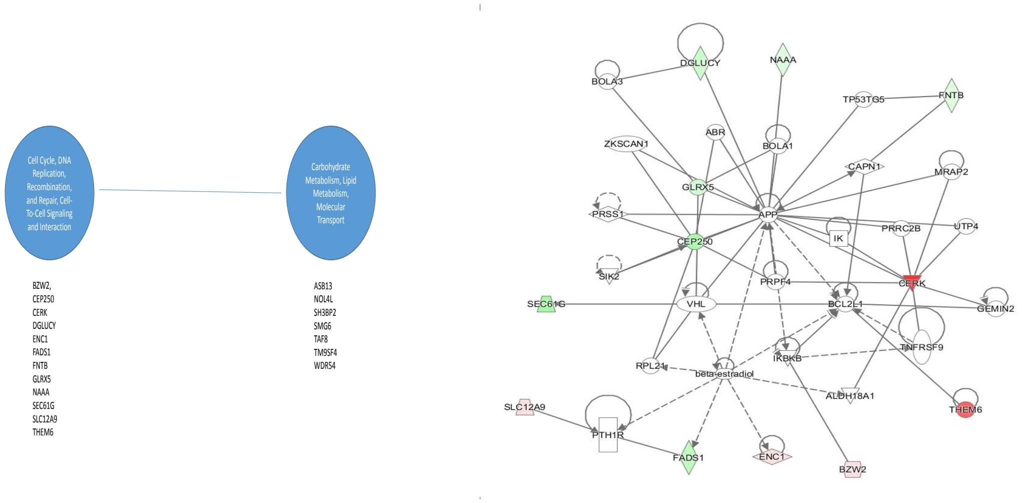

Figure S2: Subnetwork in S1 corresponding to Cancer, Cell Death and Survival, Organismal Injury and Abnormalities. Red indicates that the expression of the gene has negative impact on survival outcome and cyan indicates positive impact. White denotes no impact.

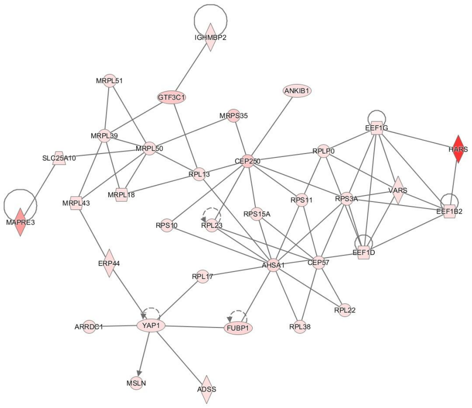

Table S1: The list of all subnetworks identified for endometrial cancer from Random Forest selection by Ingenuity Pathways Analysis. It is worth noting that all 25 networks are isolated from each other. ID: Pathway ID ranked by p-values. No. gene: number of molecules mapped in the corresponding pathway.

ID	No. gene	Top disease and Functions
1	35	RNA Post-Transcriptional Modification, Nucleic Acid Metabolism, Small Molecule Biochemistry
2	35	Cancer, Cell Death and Survival, Organismal Injury and Abnormalities
3	34	Connective Tissue Disorders, Developmental Disorder, Hereditary Disorder
4	33	Metabolic Disease, Molecular Transport, Developmental Disorder
5	33	Hereditary Disorder, Neurological Disease, Organismal Injury and Abnormalities
6	33	RNA Post-Transcriptional Modification, Cellular Assembly and Organization, Cellular Function and Maintenance
7	33	Developmental Disorder, Neurological Disease, Organismal Injury and Abnormalities
8	32	Developmental Disorder, Hereditary Disorder, Metabolic Disease
9	32	RNA Post-Transcriptional Modification, Cellular Assembly and Organization, Developmental Disorder
10	32	Hematological System Development and Function, Hematopoiesis, Humoral Immune Response
11	32	Hereditary Disorder, Neurological Disease, Organismal Injury and Abnormalities
12	31	Cellular Assembly and Organization, Cellular Compromise, Cell Cycle
13	31	RNA Post-Transcriptional Modification, Cellular Development, Skeletal and Muscular Disorders
14	31	Cancer, Neurological Disease, Organismal Injury and Abnormalities
15	30	Cellular Development, Developmental Disorder, Gastrointestinal Disease
16	30	Cell Morphology, Cellular Function and Maintenance, Organ Morphology
17	30	Lipid Metabolism, Molecular Transport, Small Molecule Biochemistry
18	30	Cancer, Dermatological Diseases and Conditions, Organismal Injury and Abnormalities
19	30	DNA Replication, Recombination, and Repair, Cell Morphology, Cellular Function and Maintenance
20	30	RNA Post-Transcriptional Modification, Lipid Metabolism, Small Molecule Biochemistry
21	30	Embryonic Development, Cardiovascular Disease, Developmental Disorder
22	29	Carbohydrate Metabolism, Lipid Metabolism, Molecular Transport
23	29	Connective Tissue Disorders, Developmental Disorder, Hereditary Disorder
24	29	Cell Signaling, Post-Translational Modification, Protein Synthesis
25	29	Organismal Development, Visual System Development and Function, Lipid Metabolism

References

[1] A. Gelman, A. Jakulin, M. G. Pittau, and Y. Su. A weakly informative default prior distribution for logistic and other regression models. *The Annals of Applied Statistics*, 2(4):1360–1383, Dec. 2008.

[2] M. D. Hoffman and A. Gelman. The no-U-turn sampler: Adaptively setting path lengths in hamiltonian monte carlo. *The Journal of Machine Learning Research*, 15(1):1593–1623, 2014.

[3] R. M. Neal. *Bayesian learning for neural networks*. PhD thesis, University of Toronto, 1995.

[4] R. M. Neal et al. Mcmc using hamiltonian dynamics. *Handbook of Markov Chain Monte Carlo*, 2(11):2, 2011.

[5] M. Plummer et al. Jags: A program for analysis of bayesian graphical models using gibbs sampling. In *Proceedings of the 3rd international workshop on distributed statistical computing*, volume 124, page 125. Vienna, 2003.
